# Antioxidant activity and phenolic profiles of the wild currant *Ribes magellanicum* from Chilean and Argentinean Patagonia

**DOI:** 10.1002/fsn3.323

**Published:** 2015-12-19

**Authors:** Felipe Jiménez‐Aspee, Samanta Thomas‐Valdés, Ayla Schulz, Ana Ladio, Cristina Theoduloz, Guillermo Schmeda‐Hirschmann

**Affiliations:** ^1^ Laboratorio de Química de Productos NaturalesInstituto de Química de Recursos NaturalesUniversidad de TalcaTalcaChile; ^2^ Laboratorio EcotonoINIBIOMA (CONICET)‐Universidad Nacional del ComahueBarilocheRío NegroArgentina; ^3^ Laboratorio de Cultivo CelularFacultad de Ciencias de la SaludUniversidad de TalcaTalcaRegión del MauleChile

**Keywords:** Antioxidant, comparative profiles, HPLC‐DAD‐MS, Patagonian currant, *Ribes magellanicum*

## Abstract

The Patagonian currant *Ribes magellanicum* is highly valued due to its pleasant flavor and sweet taste. The aim of this study was to characterize its constituents and to assess their antioxidant and cytoprotective properties. For the fruit phenolic‐enriched extract (PEE), total phenolics (TP), total flavonoids (TF), and antioxidant activity (DPPH, Ferric reducing antioxidant power (FRAP), and Trolox equivalent antioxidant activity (TEAC)) were determined. Argentinean samples presented better activity in the DPPH and FRAP assays. Best cytoprotection against oxidative stress induced by H_2_O_2_ in AGS cells was found in one Argentinean sample at 500 *μ*g mL^−1^ (65.7%). HPLC MS/MS analysis allowed the tentative identification of 59 constituents, including eight anthocyanins, 11 conjugates of caffeic‐, ferulic‐, and coumaric acid, and 38 flavonoids, most of them quercetin and kaempferol derivatives. Argentinean samples showed a more complex pattern of anthocyanins, hydroxycinnamic acids (HCA), and flavonoids. Cyanidin rhamnoside hexoside and cyanidin hexoside were the main anthocyanins, accounting for 35 and 55% for the Argentinean and 60 and 27% for the ripe Chilean fruits. HCA content was about three times higher in Argentinean samples. The phenolic profiles of Chilean and Argentinean *Ribes magellanicum* show remarkable differences in chemical composition with higher HCA and flavonoid content in Argentinean samples.

## Introduction

High intake of fruits and vegetables is inversely related to the incidence of several pathologies, including obesity (González‐Castejón and Rodriguez‐Casado 2011), age‐related neurodegenerative diseases (Shukitt‐Hale et al. 2008), cardiovascular diseases, diabetes, and certain types of cancer (Boeing et al. 2012). These health benefits have been attributed to the presence of polyphenols, especially flavonols, anthocyanins, proanthocyanidins, and phenolic acids (Liu 2003). Polyphenols possess a high antioxidant potential which may help to decrease or repair oxidative stress generated during physiological processes (Hollman 2001). Wild or cultivated berries are recognized as health‐promoting fruits due to their high content of antioxidant phytochemicals (Zafra‐Stone et al. 2007). Wild plants are often incorporated into traditional medicine or are used in a balanced diet, improving the state of well‐being and reducing the risk of diseases. Nutraceuticals are generally defined as products that promote health in addition to their nutritional value. For this reason, it is relevant to identify, characterize and quantify the phytochemicals contained in these plants.

**Figure 1:**
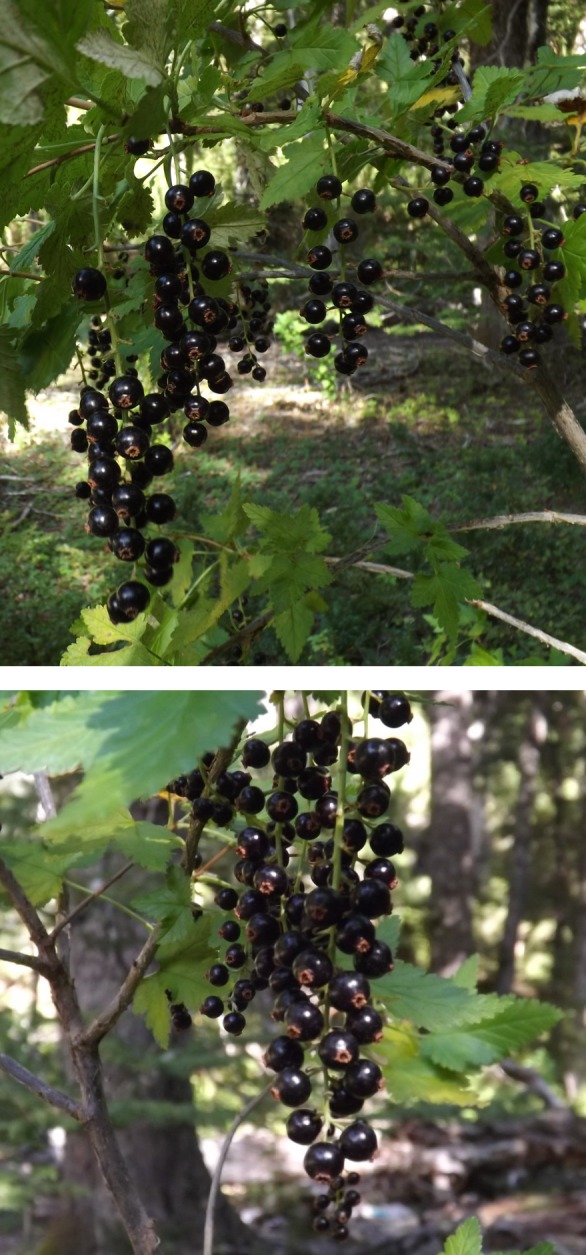
*Ribes magellanicum* ripe fruits (Parque Nacional Conguillio, Chile).

The fruits of the *Ribes* genus are gaining importance and are considered as promising crops with high economic value, especially since they are ranked second in consumer preferences after strawberries (Djordevic et al. 2010). The Patagonian region in South America provides a wide range of native plants due to the geographic and climatologic characteristics of its territory. The Patagonian wild currant (*Ribes magellanicum* Poir., Grossulariaceae) is a native species occurring in southern Chile and Argentina. This species has a long history of use among Amerindians of Patagonia and played a relevant role in their gathering for subsistence. It has a pleasant flavor and sweet taste with an intense purple color (Fig. 1). The fruits can be consumed fresh, in preserves and in syrups (Sparre 1984). In Chile and Argentina, these Patagonian currants are collected in the wild or cultivated in home gardens (Rapoport and Ladio 1999; Ladio et al. 2007; Eyssartier et al. 2011; Molares and Ladio 2012).

Scarce information has been published regarding the chemical composition and nutritional characteristics of Patagonian native berries. In *R. magellanicum* collected in southern Chile, the 3‐glucoside, and 3‐rutinoside derivatives of cyanidin are predominant among anthocyanins (Ruiz et al. 2013). Caffeoylquinic acid, feruloylquinic‐ and coumaroylquinic acids, quercetin hexoside, rutinoside and acetyl‐derivatives, kaempferol‐acetylhexoside and rutinoside derivatives, myricetin, and kaempferol were found in this berry in southern Chile (Ruiz et al. 2015). However, no information was found regarding phenolic profiling from Argentinean *R. magellanicum*.

While little is known about the wild Patagonian currant *R. magellanicum,* the chemical profiling of European currants has been extensively described, mainly for *Ribes nigrum* (black currants) and *Ribes rubrum* (red currants) (Määttä et al. 2003; Benvenuti et al. 2004; Anttonen and Karjalainen 2006; Kapasakalidis et al. 2009; Borges et al. 2010; Pinto et al. 2010; Gavrilova et al. 2011). *Ribes nigrum* contains *p*‐coumaric, ferulic, and caffeic acid derivatives (Määttä et al. 2003; Gavrilova et al. 2011), as well as myricetin, quercetin, isorhamnetin, and kaempferol derivatives as the main flavonols (Zheng et al. 2012). *Ribes rubrum* presents caffeoyl and *p*‐coumaroyl hexoside derivatives, rutin, (+)‐catechin, and epigallocatechin (Gavrilova et al. 2011). Anthocyanins comprise up to 85% of the total phenolic content, whereas hydroxycinnamic acid derivatives just represent 1–6% of the total phenols in the currants (Gavrilova et al. 2011).

There is an increasing international interest in South American native berries, mainly due to their potential health benefits (Schreckinger et al. 2010). In this context, the aim of this work was to compare the chemical composition and antioxidant activity of *Ribes magellanicum* fruits from Chilean and Argentinean Patagonia. Following our studies on South American food plants, we now report the phenolic, flavonoid, and anthocyanin content and composition, antioxidant activity, and cytoprotection toward oxidative stress, as well as polyphenol profiling in *R. magellanicum*.

## Materials and Methods

### Chemicals

Folin‐Ciocalteu phenol reagent, 2,4,6‐tri(2‐pyridyl)1,3,5‐triazine (TPTZ), potassium chloride, sodium acetate, 1,1‐diphenyl‐2‐picrylhydrazyl radical (DPPH), quercetin, gallic acid, L‐glutamine, MTT, AlCl_3_, and hydrogen peroxide were purchased from Sigma‐Aldrich (St. Louis, MO). 2,2′‐Azino‐bis(3‐ethylbenzothiazoline‐6‐sulfonic acid) diammonium salt (ABTS), 6‐hydroxy‐2,5,7,8‐tetramethylchroman‐2‐carboxylic acid (Trolox), potassium persulfate, sodium carbonate, sodium bicarbonate, acetic acid, FeCl_3_.6H_2_O, HPLC‐grade methanol, and formic acid were purchased from Merck (Darmstadt, Germany). Quercetin‐3‐glucoside (99.14%), kaempferol‐3‐glucoside (99.37%), quercetin‐3‐glucuronide (95.91%), cyanidin‐3‐glucoside (99.21%), cyanidin‐3‐rutinoside (98.02%), delphinidin‐3‐glucoside (98.02%), *trans*‐caffeic acid (99.81%), and 3‐caffeoylquinic acid (98.95%) were from PhytoLab GmbH (Vestenbergsgreuth, Germany). Ultrapure water was obtained using a Barnsted EasyPure water filter (Thermo Scientific, Marietta, OH). Culture medium Ham F‐12, penicillin, streptomycin, and fetal bovine serum (FBS) were obtained from Invitrogen Corp. Chemicals (Grand Island, NY).

### Fruit material and sample preparation

**Figure 2:**
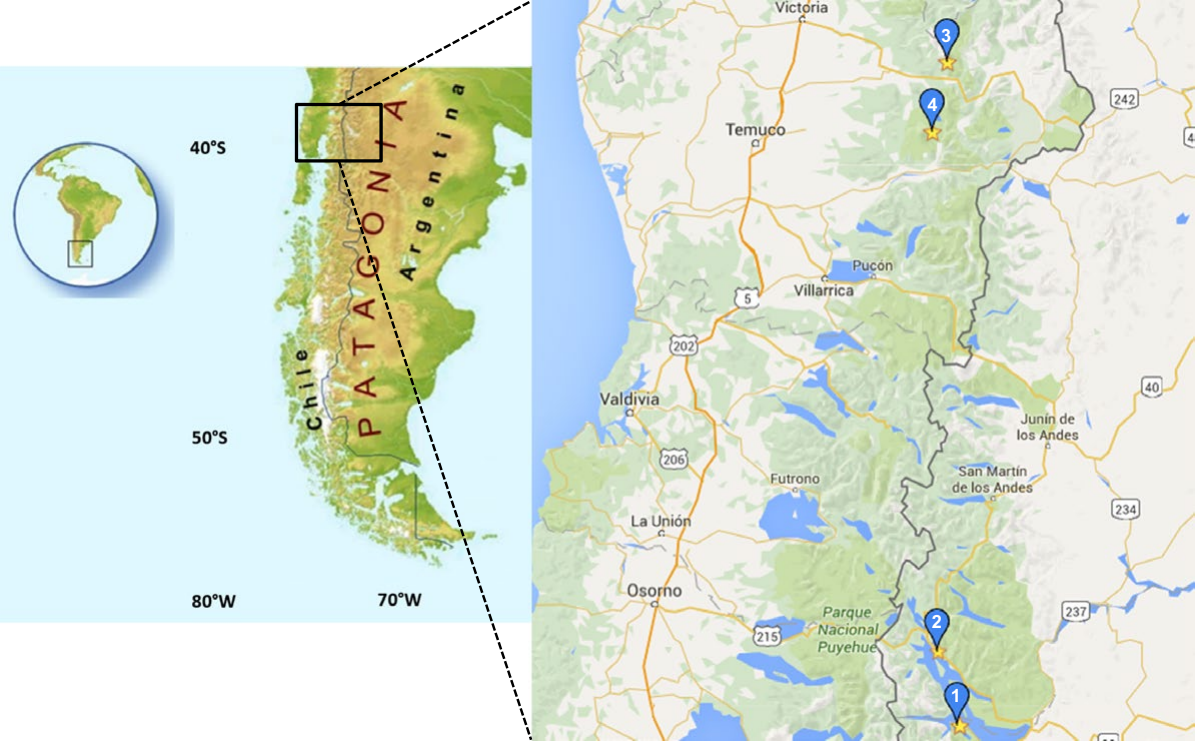
Map of Patagonia showing the geographic collection places of *Ribes magellanicum* fruits. Argentina: (1) Arroyo Casa de Piedra, (2) Laguna Verde, Villa La Angostura (Parque Nacional Nahuel Huapi). Chile: (3) Reserva Nacional Malalcahuello, (4) Parque Nacional Conguillio. Source: Google maps.

Argentinean fruits were collected at the Provincia de Rio Negro, Arroyo Casa de Piedra (41°07′07″S; 71°27′56″W) (ripe fruits) (1) and Laguna Verde, Villa La Angostura (40°45′55″S; 71°38′41″W) (turning fruits) (2). Chilean fruits were collected at the Region de La Araucanía, Parque Nacional Conguillio (39°29′36′'S; 71°41′53′'W) (ripe fruits) (3) and Reserva Nacional Malalcahuello (38°25′13′'S; 71°36′48′′W) (turning fruits) (4). The collection places are shown in Figure 2. The samples from Chile were identified by Prof. Patricio Peñailillo (Universidad de Talca herbarium), whereas samples from Argentina by Prof. Ana Ladio, Universidad Nacional del Comahue.

Fruits were homogenized in a blender and extracted four times with MeOH:formic acid (99:1 v/v) in a 1:3 v/v homogenate:solvent ratio in the dark under sonication (15 min each). The combined extracts were dried under reduced pressure at 37°C. To obtain the phenolic‐enriched extract (PEE), the samples were adsorbed into Amberlite XAD‐7 resin. The Amberlite resin was preconditioned as follows: the resin was washed with 0.1N NaOH, rinsed with distilled water, washed with 0.1N HCl and rinsed with distilled water to remove acid, and stored with MeOH:H_2_O (1:9 v/v) until use. The concentrated MeOH extract was dissolved in water, filtrated and mixed with Amberlite XAD‐7 in a 1:5 (extract:Amberlite) ratio and continuously stirred for 40 min. The resin was filtered, washed with water and phenolics were desorbed with MeOH. The MeOH solution was evaporated under reduced pressure at 40°C and freeze‐dried for analysis, obtaining the phenolic enriched extract (PEE). The accuracy of the extraction method was evaluated by spiking a fruit sample with standard solutions of chlorogenic acid (78.9 mg Kg^−1^), cyanidin‐3‐rutinoside (6.9 mg Kg^−1^), and kaempferol‐3‐glucoside (6.9 mg Kg^−1^). The recovery percentages of the standards were calculated by means of HPLC‐DAD.

### Total phenolic and total flavonoid content

The TP content was determined by the Folin–Ciocalteu method with slight modifications (Jiménez‐Aspee et al. 2014). Stock solutions of 5 g L^−1^ were prepared in MeOH:H_2_O 1:1. For TP, the results are expressed as grams of gallic acid equivalents per kilogram PEE (g GAE kg^−1^). The TF content was determined as described by Simirgiotis et al. (2013). For TF, results are expressed as grams of catechin equivalents per kilogram PEE (g CatE kg^−1^).

### Scavenging of the 2,2‐diphenyl‐1‐picryl hydrazyl

The free radical‐scavenging activity of the samples was determined by the discoloration of the free radical DPPH (20 mg L^−1^) as previously described (Jiménez‐Aspee et al. 2014). Extracts were dissolved in MeOH: H_2_O (1:1) to a final concentration of 300 mg L^−1^. Serial dilutions of the stock solutions of each sample were prepared in 96‐well plates. Methanol was used as the negative control and quercetin as the reference compound. Absorbance was measured at 517 nm in a universal microplate reader (Biotek Instruments Inc., ELx 800, Winooski, VT). The concentration of the extract that scavenges the DPPH radical by 50% (SC_50_) is expressed as milligrams of PEE per liter of solution (mg L^−1^) and was calculated using the OriginPro 8.0 software (OriginLab Corporation, Northampton, MA).

### Ferric reducing antioxidant power

The FRAP assay was performed as previously described (Simirgiotis et al. 2013; Jiménez‐Aspee et al. 2014). Samples were dissolved in MeOH:H_2_O (1:1) to a final concentration of 300 mg L^−1^. Absorbance was read at 593 nm using a Thermo Spectronic Helios Alfa spectrophotometer (Cambridge, U.K.). Quantification was performed using a standard curve of the antioxidant Trolox. Results are expressed as millimol Trolox equivalents per kilogram of PEE (mmol TE kg^−1^).

### Trolox equivalent antioxidant activity

ABTS radical‐scavenging activity was determined according to Nenadis et al. (2004). The ABTS* was generated by mixing ABTS solution with potassium persulfate. The solution was stored in the dark for 16 h, and diluted with methanol to a final absorbance of 0.700 ± 0.050 at 734 nm. Determinations were carried out by mixing ABTS* with fresh standard (1 mmol L^−1^ Trolox) or PEE (100, 150, 200, 250, and 300 mg L^−1^). Absorbance was read at 734 nm after 6 min. Results are expressed as *μ*mol Trolox equivalents per gram of PEE (*μ*mol L^−1^ TE g^−1^).

### AGS cell culture

Human epithelial gastric cells AGS (ATCC CRL‐1739) were grown in Ham F‐12 medium supplemented with 1 mmol L^−1^ l‐glutamine, 1.5 g L^−1^ sodium bicarbonate, 100 g L^−1^ heat‐inactivated fetal bovine serum (FBS), 100 IU mL^−1^ penicillin, and 100 mg L^−1^ streptomycin. Cells were grown as monolayers in a humidified incubator with 5% CO_2_ in air at 37°C.

### Cytotoxicity assay

Cytotoxicity values (IC_50_, *μ*g mL^−1^) were required as a reference to determine the working concentrations of the PEEs in the cytoprotection experiment. Confluent cultures of AGS cells were treated during 24 h with the medium containing the lyophilized PEE samples at concentrations ranging from 0 up to 1000 mg L^−1^. The samples were dissolved in the medium supplemented with 20 g L^−1^ FBS. Cells treated with the medium only were used as controls. Cell viability was determined at the end of the incubation by means of the MTT reduction assay (Cheli and Baldi 2011), and the concentration that inhibits 50% of cell viability was calculated (IC_50_).

### Cytoprotection against H_2_O_2_‐induced stress

The cytoprotective activity of the extract was evaluated against H_2_O_2_‐induced stress, following the previously reported methodology (Jimenez‐Aspee et al. 2016). Briefly, confluent cultures of AGS cells were treated overnight with different concentrations of *Ribes magellanicum* PEEs (0, 62.5, 125, 250, and 500 mg L^−1^). Extracts were dissolved in the medium supplemented with 20 g L^−1^ FBS and antibiotics. At the end of the incubation, culture medium was completely removed via vacuum aspiration. Then, oxidative stress was induced with 442 mg L^−1^ H_2_O_2_ for 2 h. The H_2_O_2_ solution was prepared using the medium without any supplements. Untreated cells were used as 100% viability controls. Cells treated with H_2_O_2_ only served as damage control. Quercetin was used as the reference compound (Heo et al. 2008). After the oxidative stress challenge, cell viability was determined by means of MTT reduction assay (Cheli and Baldi 2011). Each concentration was tested in quintuplicate and experiments were repeated twice. Results are expressed as a percentage of the viability control.

### HPLC‐DAD analysis

The PEEs were analyzed by HPLC coupled to a diode array detector to compare the samples and set the conditions for HPLC‐DAD‐MS/MS studies. The HPLC system used for DAD analysis was a Shimadzu equipment (Shimadzu Corporation, Kyoto, Japan) consisting of a LC‐20AT pump, a SPD‐M20A UV diode array detector, CTO‐20AC column oven and a LabSolution software. A MultoHigh 100 RP 18‐5 *μ* (250 × 4.6 mm) column (CS‐Chromatographie Service GmbH, Langerwehe, Germany) maintained at 25 °C was used. The HPLC analyses were performed using a linear gradient solvent system consisting of 1% formic acid in water (A) and acetonitrile (B) as follows: 90% A to 75% A over 30 min, followed by 75% A to 90% A from 30 to 50 min, and finalizing with 90% A for 5 min. The flow rate was 1 mL min^−1^ and the volume injected was 20 *μ*L. The compounds were monitored at 254, 320, 350, and 520 nm and spectra from 200 to 600 nm were recorded for peak characterization. The results of the HPLC‐DAD chromatograms were compared with the HPLC‐MS analysis. The compounds were identified based on the elution profile, retention time, UV, and mass spectra.

### Quantification of main anthocyanins, hydroxycinnamic acids, and flavonols

Quantification of the main phenolics was carried out by HPLC‐DAD using external calibration curves prepared with pure standards. For anthocyanins, two curves were prepared using the main anthocyanins occurring in the sample: cyanidin‐3‐glucoside (12.5–800 mg L^−1^, *r* = 0.9962) and cyanidin‐3‐rutinoside (12.5–800 mg L^−1^, *r* = 0.9999) and were measured at 520 nm. For hydroxycinnamic acids (HCA), a curve with 3‐caffeoylquinic acid (10‐800 mg L^−1^, *r* = 0.9990) was carried out and measured at 320 nm. For flavonols, two curves were prepared using quercetin‐3‐glucoside (5–80 mg L^−1^, *r* = 0.9987) and kaempferol‐3‐glucoside (5–80 mg L^−1^, *r* = 0.9997), and measured at 350 nm. The samples were injected at 5 g L^−1^ twice and the peak areas at 320, 350, and 520 nm were determined. The peak areas were quantified through comparison with the corresponding calibration curve and results are expressed as mg equivalents of the corresponding standard per kg of PEE. For comparative purposes, an internal standard was added to samples in parallel before HPLC analysis. For HCA the internal standard was *trans*‐caffeic acid (100 mg L^−1^), for flavonols the internal standard was quercetin‐3‐glucuronide (40 mg L^−1^), and for anthocyanins the internal standard was delphinidin‐3‐glucoside (100 mg L^−1^). Previous HPLC analyses of the extracts were carried out to establish that these compounds were not detectable in the PEE.

### Identification of phenolics by HPLC‐ESI‐MS/MS

Mass spectra were recorded using an HPLC HP1100 (Agilent Technologies Inc., Santa Clara, CA) liquid chromatography system connected through a split to an Esquire 4000 Ion Trap LC/MSystem (Bruker Daltoniks, Germany). Full‐scan mass spectra were measured between *m/z* 150 and 2000 *μ*. Mass spectrometry data were acquired in the negative mode for all phenolic compounds except anthocyanins, which was acquired in the positive ion mode. Nitrogen was used as nebulizer gas at 27.5 psi, 350 °C and at a flow rate of 8 L min^−1^. The mass spectrometric conditions were as follows: electrospray needle, 4000 V; end plate offset, −500 V; skimmer 1, 56.0 V; skimmer 2, 6.0 V; capillary exit offset, 84.6 V; and capillary exit, 140.6 V. Collision‐induced dissociation (CID) spectra were obtained with a fragmentation amplitude of 1.00 V (MS/MS) using helium as the collision gas.

### Statistical analysis

Determinations of TP, TF, DPPH, and FRAP were performed in triplicate and results are expressed as mean values ± SD. For the TEAC assay, a curve was plotted for each sample and a correlation coefficient with 95% confidence limit was established. Experiments with AGS cells were carried out twice using different cell preparations. Each concentration was tested in quintuplicate. Statistical differences between different treatments and their respective control were determined by one‐way analysis of variance (ANOVA) and Dunnett's multiple comparison test. The level of significance was set at *P *<* *0.05. The Spearman's correlation coefficient for nonparametric data with a significance level of *P *≤* *0.05 was used to determine the relationships among variables. Additionally, a multivariate statistical method was applied to the data set. The chemical descriptors for *Ribes* samples were: TP, TF, quantified phenolics, DPPH, and FRAP. Analyses were carried out using the software SPSS 14.0 (IBM, Armonk, NY) and the statistical package Statistica 12.7.207.0 (Dell Inc., Tulsa, OK).

## Results and Discussion

Four *R. magellanicum* collections, including samples from Argentina and Chile, were assessed for TP and TF content, DPPH, FRAP, and TEAC antioxidant activity, cytoprotective effect against oxidative stress‐induced toxicity, and phenolic composition. The PEE yield in terms of fresh fruit was as follows (in g kg^−1^): Arroyo Casa de Piedra: 8.5; Laguna Verde, Villa La Angostura: 6.2; Parque Nacional Conguillio: 17.3; Reserva Nacional Malalcahuello: 3.0. The recovery of phenolic compounds was 117.21% for chlorogenic acid, 85.16% for cyanidin‐3‐rutinoside and 78.44% for kaempferol‐3‐glucoside. According to Burin et al. (2011) these ranges of recovery would indicate that the extraction method used was acceptable for the quantification of these compounds.

### Total phenolic, total flavonoid content, and antioxidant activity

The TP and TF values were higher for ripe Argentinean fruits (320.0 g GAE kg^−1^ and 164.0 g CatE kg^−1^ compared with 117.0 g GAE kg^−1^ and 48.6 g CatE kg^−1^ for the ripe Chilean sample, respectively) (Table 1). When comparing Argentinean and Chilean *R. magellanicum*, samples showed large differences regarding antioxidant activity (Table 1). In the Argentinean collection of Arroyo Casa de Piedra, DPPH, FRAP, and TEAC values were of 6.2 mg L^−1^, 4006.3 mmol TE kg^−1^ PEE, and 2856.9 *μ*mol L^−1^ TE g^−1^ PEE, respectively. In the Chilean fruits, the best activity was found in the Parque Nacional Conguillio sample with a SC_50_ of 22.9 mg L^−1^ in the DPPH, 1182.4 mmol TE kg^−1^ PEE in the FRAP, and 1577.7 *μ*mol L^−1^ TE g^−1^ PEE in the TEAC assay. According to Ruiz et al. (2013), the antioxidant effect from *R. magellanicum* was 64.1 *μ*mol TE g^−1^ fresh fruit in the TEAC assay, whereas in the CUPRAC assay the value was 71.16 *μ*mol TE g^−1^ fresh fruit (Ruiz et al. 2015). Among the Chilean Patagonian berries *R. magellanicum* showed the best CUPRAC activity and one of the best TEAC activities (Ruiz et al. 2013).

**Table 1:** Total phenolics (TP), total flavonoids (TF), and antioxidant activity of phenolic enriched extract (PEEs) from *Ribes magellanicum* from Argentina and Chile

Samples	TP (g GAE kg^−1^ PEE)	TF (g CatE kg^−1^ PEE)	Antioxidant activity		
			DPPH (SC_50_ in mg PEE L^−1^)	FRAP (mmol TE kg^−1^ PEE)	TEAC (*μ*mol L^−1^ TE g^−1^ PEE)
Argentina					
Arroyo Casa de Piedra	320.0 ± 0.0	164.0 ± 1.7	6.2 ± 0.3	4006.3 ± 106.5	2856.9
Laguna Verde, Villa La Angostura	59.0 ± 1.0	56.8 ± 0.3	10.6 ± 0.4	1616.3 ± 22.5	1414.3
Chile					
Parque Nacional Conguillio	117.0 ± 1.0	48.6 ± 0.3	22.9 ± 1.2	1182.4 ± 22.5	1577.7
Reserva Nacional Malalcahuello	48.0 ± 1.0	31.4 ± 0.6	24.2 ± 0.5	816.2 ± 15.0	1098.7

GAE, gallic acid equivalent; CatE, catechin equivalent; DPPH, diphenyl picryl hydrazyl radical; FRAP, ferric reducing antioxidant power; TEAC, Trolox equivalent antioxidant capacity; TE, Trolox equivalent. All determinations were carried out in triplicate and results are expressed as mean values ± SD.

### Cytoprotection against chemical oxidative damage

**Figure 3:**
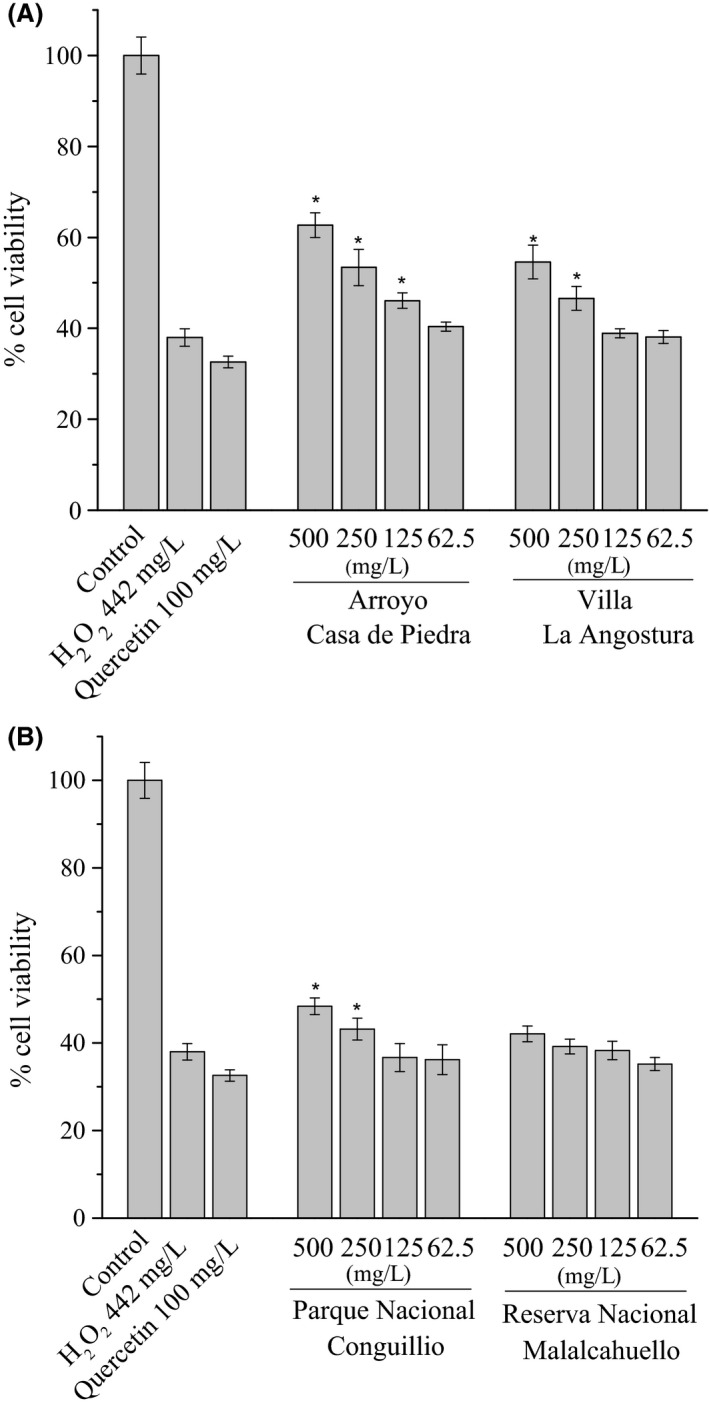
Cytoprotective effect of *Ribes magellanicum* PEEs against H_2_O_2_‐induced oxidative stress on AGS cells. (A) Argentinean samples, (B) Chilean samples. The cell viability was determined by MTT reduction assay. Results are expressed as means ± SD (*n* = 5). *P <* *0.05 compared to H_2_O_2_ controls.

Under our assay conditions, PEEs were devoid of toxicity toward AGS cells, with IC_50_ values >1000 mg L^−1^. In MRC‐5 cells a 10,000 mg L^−1^ *Ribes nigrum* extract concentration induced 20% decrease in cell viability and was selected for further experiments (Jia et al. 2014). We selected 500 mg L^−1^ of PEE for the following studies as the highest concentration. *Ribes magellanicum* PEEs showed a significant cytoprotective dose‐dependent effect against oxidative damage induced by H_2_O_2_ in AGS cells. Hydrogen peroxide (442 mg L^−1^) caused a significant decrease in cell viability (57.3%) compared to untreated controls. Exogenous H_2_O_2_ can enter cells inducing cytotoxicity due to its high membrane permeability, and through Fenton reaction can form highly reactive ·OH species causing oxidative injury in cells (Halliwell et al. 1992; Halliwell 1994). The positive control quercetin (100 mg L^−1^) did not protect cells from oxidative damage. From the Argentinean samples, Arroyo Casa de Piedra showed significant protective effect at 125 mg L^−1^ (20.0%), 250 mg L^−1^ (42.9%), and 500 mg L^−1^ (65.7%), compared with the H_2_O_2_ control (Fig. 3A). The sample from Villa La Angostura presented significant activity at 250 mg L^−1^ (22.9%) and 500 mg L^−1^ (42.9%) (Fig. 3A). From the Chilean samples, only the PEE from Conguillio presented significant activity at 250 mg L^−1^ (15.2%) and 500 mg L^−1^ (27.3%) (Fig. 3B).

Anthocyanins and other flavonoids may exert their protective antioxidant effects in humans before absorption into the systemic bloodstream by directly scavenging free radicals (Moskaug et al. 2005), thus protecting cells of the gastrointestinal tract (Halliwell et al. 2005). Black currant phenolics have previously demonstrated a protective effect against H_2_O_2_‐induced oxidative stress in human fetal lung fibroblast MRC‐5 cells (Jia et al. 2014), neuroblastoma SH‐SY5Y human promyelocytic leukemia cells, and gastric cancer SGC‐7901 cells (Ghosh et al. 2006). The authors attributed the improved viability of cells against H_2_O_2_ to the neutralization capabilities of black currant extracts against radical oxidants. Moreover, the extract was responsible for up‐modulation of the antioxidant enzymes superoxide dismutase, catalase, and glutathione peroxidase (Jia et al. 2014). In a recent study on another Patagonian berry, *Rubus geoides*, the fruit extract increased the intracellular GSH content and protected AGS cells against oxidative and dicarbonyl‐induced stress (Jimenez‐Aspee et al. 2016). In a study on the protective effect of *Rubus coreanus* on PC‐12 cells against H_2_O_2_, the authors determined that cyanidin glycosides present in the fruit effectively diminished intracellular oxidative stress, whereas the nonanthocyanin fraction of the extract had no significant effect (Im et al. 2013). Our results show that the Argentinean samples were the most effective in preventing oxidative damage in human gastric epithelial AGS cells.

### HPLC‐DAD and HPLC‐MS/MS analysis

**Figure 4:**
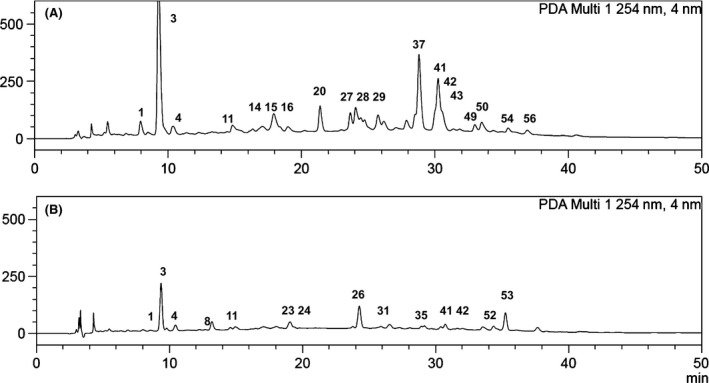
Representative HPLC‐DAD chromatograms of *Ribes magellanicum* PEE at 254 nm. (A) Arroyo Casa de Piedra, Argentina; (B) Parque Nacional Conguillio, Chile. Peak numbers refer to Table 2.

**Figure 5:**
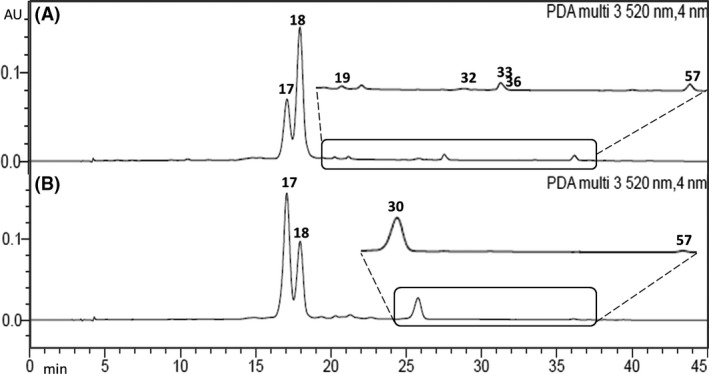
Representative HPLC‐DAD chromatograms of *Ribes magellanicum* PEE at 520 nm. (A) Arroyo Casa de Piedra, Argentina; (B) Parque Nacional Conguillio, Chile. Peak numbers refer to Table 2.

All PEEs were compared by HPLC‐DAD to get an insight into the chemical diversity of *R. magellanicum* fruits and to disclose similarities and differences in the composition according to the collection place and ripening stage. The HPLC chromatograms (at 254 nm) of ripe Argentinean and Chilean fruits are presented in Figure 4, whereas Figure 5 shows the HPLC trace of anthocyanins detected at 520 nm. HPLC MS/MS analysis allowed the tentative identification of 59 constituents, including anthocyanins, hydroxycinnamic acids, and flavonols. The tentative identification of the compounds is summarized in Table 2.

**Table 2:** Tentative identification of phenolics in *Ribes magellanicum* fruits from Argentina and Chile by HPLC‐DAD‐ESI‐MS

Peak	Rt (min)	*λ* max (nm)	MW	[M + H]^+^ or [M − H]^−^	+/− ions	MS/MS	Tentative identification
**1**	8.4	324, 300, 238	354	353	−	191, 179	Caffeoyl quinic acid
**2**	8.5	324, 290 sh, 238	730	729	−	353, 179	Caffeoyl quinic acid derivative
**3**	8.8–8.9	325, 290 sh, 239	354	353	−	191, 179, 135	3‐Caffeoyl quinic acid^a^
**4**	9.8	330, 215	180	179	−	134	Caffeic acid derivative
**5**	10.3		342	341	−	179	Caffeoyl hexoside
**6**	10.5	320 sh 281, 240	354	353	−	191, 179	Caffeoyl quinic acid isomer
**7**	10.9		290	289	−	245	Catechin/Epicatechin
**8**	12.6		342	341	−	179	Caffeoyl hexoside
**9**	13.7		464	463	−	417, 301	Q‐hexoside
**10**	14.3	330 sh, 281, 240	354	353	−	191, 179, 173	Caffeoyl quinic acid isomer
**11**	14.6		368	367	−	193	Feruloyl quinic acid
**12**	16.0		368	367	−	193	Feruloyl quinic acid
**13**	15.9–16.1		448	447	−	285	K‐hexoside
**14**	16.1		594	593	−	285	K‐rhamnosidehexoside
**15**	16.4		466	465	−	285	K‐derivative
**16**	16.8		594	593	−	447, 285	K‐hexosiderhamnoside
**17**	16.4–17.3	517, 280	593	594	+	449, 287	Cyanidin rhamnosyl hexoside
**18**	18.7	517, 280	450	449	+	287	Cyanidin glucoside
**19**	20.1		466	465	+	303	Delphinidin hexoside isomer
**20**	20.6–20.8	352, 300 sh, 272	770	771	−	609, 591, 505, 301	Q‐dihexosylrhamnoside
**21**	21.0		776	775	−	467, 313,163	Coumaroyl hexoside rhamnoside derivative
**22**	21.1		449	449	−	285	K derivative
**23**	21.3		626	625	−	463, 301	Q‐dihexoside
**24**	21.5		610	609	−	463, 316	Myr dipentoside
**25**	21.9		742	741	−	609, 591, 475, 301	Q‐pentoside derivative
**26**	22.3	353, 300 sh, 270	610	609	−	463, 301	Q‐hexosiderhamnoside
**27**	23.1		626	625	−	463, 301	Q‐dihexoside
**28**	24.1		656	625	−	316	Myr hexoside pentoside
**29**	24.6		480	479	−	316, 179	Myr hexoside
**30**	24.6		478	477	+	287	Cyanidin derivative
**31**	25.4–25.6		610	609	−	463, 343, 301	Q‐rhamnoside hexoside
**32**	25.5		620	619	+	487, 317	Petunidin derivative
**33**	27.8		952	951	+	634, 557, 476, 331	Malvidin rhamnoside derivative
**34**	28.0–28.3	353, 300 sh, 270	610	609	−	463, 301	Q‐hexoside rhamnoside
**35**	28.1–28.4		464	463	−	316, 179	Myr pentoside
**36**	28.1		612	611	+	465, 303	Delphinidin rhamnosyl hexoside
**37**	28.5		464	463	−	301	Q‐hexoside
**38**	29.1–29.2	348, 300 sh, 296	566	565	−	433, 301	Q‐dipentoside
**39**	29.3–29.4		610	609	−	301	Q‐hexoside rhamnoside
**40**	29.5		915	914	−	895, 771, 447, 284	K‐trihexoside conjugate
**41**	29.5–29.6	348, 297	448	447	−	285	K‐hexoside
**42**	29.7		464	463	−	301	Q‐hexoside
**43**	29.8–29.9		580	579	−	447, 429, 285	K‐pentoside hexoside
**44**	30.4		594	593	−	447, 285	K‐rhamnoside hexoside
**45**	30.4–30.5		610	609	−	447, 285	K‐dihexoside
**46**	30.5		652	651	−	593, 301	Q‐conjugate
**47**	30.8		506	505	−	301	Q‐hexoside monoacetate
**48**	32.0	344, 270	594	593	−	285	K‐coumaroyl hexoside
**49**	32.5		478	477	−	433, 315	Isorhamnetin hexoside
**50**	32.9	333, 267	434	433	−	301	Ellagic acid pentoside
**51**	33.5		550	549	−	399, 285	K‐conjugate
**52**	33.7		464	463	−	301	Q‐hexoside
**53**	34.5		448	447	−	301	K‐hexoside
**54**	34.7		432	431	−	269	Apigenin hexoside
**55**	35.9		490	489	−	285	K‐hexoside monoacetate
**56**	36.1		418	417	−	285	K‐pentoside
**57**	36.3	517, 280	818	817	+	655, 449, 369, 287	Cyanidin hexoside derivative
**58**	43.1–43.2		286	285	−		K
**59**	43.5		302	301	−	178, 151	Q

K, Kaempferol; Q, Quercetin; Myr, Myricetin.

a According to Clifford et al. (2003).

### Anthocyanins

Two main anthocyanins were observed in the HPLC‐DAD chromatograms of the ripe fruits (Fig. 5), showing UV maxima of 517 nm, in agreement with cyanidin derivatives. Positive ion MS allowed the identification of several minor constituents based on the selected ion chromatograms and MS/MS data. The main anthocyanins **17** and **18,** as well as compound **30** and **57,** showed in the MS/MS spectra a *m/z* ion at 287 amu, indicative of cyanidin. The compound **17** losses a deoxyhexose (146 amu) and a hexose (162 amu), whereas **18** showed the loss of 162 amu (hexose), in agreement with cyanidin rhamnosyl hexoside and cyanidin hexoside, respectively. The compound **57** showing the loss of hexose and additional fragments leading to cyanidin was tentatively identified as cyanidin hexoside derivative, whereas compound **30** losses 190 amu leading to cyanidin and was assigned as cyanidin derivative. Compounds **19** and **36** were minor constituents presenting in the MS/MS spectra a *m/z* ion at 303 amu, in agreement with delphinidin derivatives. The compound **19** shows the loss of 162 amu (hexose), whereas **36** losses a deoxyhexose (146 amu) and a hexose (162 amu). The compounds were assigned as delphinidin hexoside and delphinidin rhamnosyl hexoside, respectively. The MS^2^ of compounds **32** and **33** shows a *m/z* ion at 317 and 331 amu, confirmed by selected ion chromatogram, in agreement with petunidin and malvidin derivatives, respectively. In *R. nigrum* four major anthocyanins distributed between cyanidin and delphinidin derivatives are responsible for more than 97% of total anthocyanin content (Slimestad and Solheim 2002) and are major contributors to the antioxidant activity (Borges et al. 2010). In *R. magellanicum* from southern Chile, Ruiz et al. (2013) reported the occurrence of delphinidin 3‐glucoside and 3‐rutinoside as minor anthocyanins and cyanidin 3‐glucoside and 3‐rutinoside as main compounds.

### Hydroxycinnamic acids

Eleven hydroxycinnamic acid derivatives were detected in the *R. magellanicum* samples, including eight caffeic‐, two ferulic‐, and one coumaric acid conjugates. Selected ion chromatograms allowed clear identification of caffeoyl, coumaroyl, and feruloyl derivatives based on the *m/z* ions at 179, 163, and 193 amu, respectively. Compounds **1**,** 3**,** 6,** and **10** presented UV spectra compatible with caffeoylquinic acid/chlorogenic acid and a pseudomolecular ion of *m/z* 353 with product ions at 191 and 179 amu, respectively, and were characterized as caffeoylquinic acid isomers. Compound **3** was the main caffeoylquinic acid in all the samples, presenting MS^1^ of 353, MS^2^ 191 and 135, and MS^3^ of 172 and 127, compatible with 3‐caffeoylquinic acid (Clifford et al. 2003). Compound **4** was identified as caffeic acid and compounds **5** and **8**, with a pseudomolecular ion at *m/z* 341 and neutral loss of 162 amu, were assigned as caffeic acid hexosides. Both compounds differ either in the identity of the hexose or in the place of attachment of caffeic acid in the sugar moiety. The compound **2** with an UV spectrum compatible with a caffeoylquinic acid derivative shows a pseudomolecular ion of *m/z* 729 amu and fragments at 353 and 179 amu, compatible with a caffeoylquinic acid derivative. Compounds **11** and **12**, with a [M − H]^−^ of 367 amu showed the neutral loss of 174 amu, leading to deprotonated ferulic acid and were assigned as feruloylquinic acid isomers. The coumaroyl hexoside pentoside derivative **21** was tentatively identified on the basis of the loss of 308 amu from the a [M − H]^−^ pseudomolecular ion at 775 amu, leading to further fragments at 467, 313, and 163 amu, compatible with a conjugate of coumaric acid.

Four caffeoylquinic acid isomers, caffeoyl hexoside, two coumaroylquinic acids and two feruloylquinic acids were detected in a southern Chile (Patagonia) collection of *R. magellanicum* (Ruiz et al. 2015). The samples investigated in our work showed caffoylquinic acid as a main phenolic and 11 hydroxycinnamic acid derivatives, most of them related to caffeic acid.

### Flavonol derivatives

The UV spectra of the main flavonoids in the samples shows maxima at 352‐353 nm for compounds **20**,** 26,** and **34** and 344–348 nm for compounds **38**,** 41,** and **48**, suggesting the occurrence of glycosides of quercetin (Q) and quercetin or kaempferol (K), respectively.

Selected ion chromatograms and MS/MS experiments led to a base peak at *m/z* 301 or *m/z* 285, allowing the identification of the compounds into derivatives of Q or K, respectively. In the samples, 38 flavonoids were tentatively identified based on the MS data and fragmentation patterns. Compounds **13–16**,** 22**,** 40**,** 41**,** 43–45**,** 48**,** 51**,** 53**,** 55**,** 56** were assigned as K conjugates and **58** was identified as the aglycone K. The K derivatives **13**,** 14**,** 16**,** 41**,** 43**,** 44**,** 45**,** 53**,** 55,** and **56** differ in the number and identity of the conjugate. Compounds **13, 41,** and **53** show neutral loss of a hexose, **14** presents the consecutive losses of rhamnose and hexose, **16** a hexose and a rhamnose, **43** shows neutral loss of a pentose and a hexose, **44** losses a rhamnose and a hexose, **45** losses two hexoses, **55** an acetate and a hexose and **56** a pentose, leading to K. The compounds were assigned as K‐hexoside, K‐rhamnoside hexoside, K‐hexoside rhamnoside, K‐pentosidehexoside, K‐rhamnosidehexoside, and K‐dihexoside, respectively. Compounds **15** and **22** show neutral loss of a 180 amu and 164 amu fragment, respectively, whereas **51** loss 264 amu leading to the *m/z* peak at 285 amu of K. Compound **40** losses three hexoses and an additional fragment and were assigned as K ‐hexoside and K‐trihexoside conjugate. The compound **48** showing the loss of a coumaroyl and hexose was assigned as K‐coumaroylhexoside. Compounds **9**,** 20**,** 23**,** 25–27**,** 31**,** 34**,** 37–39**,** 42**,** 46**,** 47**,** 52** were tentatively identified as Q glycosides and **59** was identified as Q. All of them showed the characteristic ion of Q at *m/z* 301, which further fragmented to the diagnostic peaks at 178 and 151 amu in the MS^2^ spectra. The identity of the sugars was determined by the neutral loss of hexose (162 amu), pentose (132 amu), or rhamnose (146 amu). The Q glycosides comprised a triglycoside (dihexosylrhamnoside **20**), several disaccharides including: dihexosides (**23** and **27**), hexosiderhamnoside (**26**,** 34**,** 39**), rhamnoside hexoside (**31**), dipentoside (**38**), monosaccharides (hexoside **9**,** 37**,** 42,** and **52**), a hexoside monoacetate (**47**), and the Q conjugate **46**. Four myricetin derivatives were found in *R. magellanicum*, including the dipentoside **24**, hexosylpentoside **28**, hexoside **29,** and pentoside **35**. Ruiz et al. (2015) reported 11 flavonoid glycosides from a single collection of *R. magellanicum* from Magallanes, Chilean Patagonia. The glycosides included quercetin derivatives (seven compounds), kaempferol, and myricetin rutinoside and hexoside. This new study offers a much broader array of compounds, including 38 flavonol conjugates distributed into quercetin (16), kaempferol (16), myricetin (4), isorhamnetin (1), and apigenin (1) derivatives.

### Quantification of main anthocyanins, hydroxycinnamic acids, and flavonols

Phenolic metabolites in plants are greatly influenced by environmental conditions, and their composition and content defines the quality and flavor characteristics of fruits (Mikulic‐Petkovsek et al. 2015). The location and year of collection has been shown to be an important factor affecting the composition of phenolic compounds in *Ribes nigrum* (Anttonen and Karjalainen 2006; Vagiri et al. 2013). Several strategies are reported in literature to quantify the main phenolic compounds, including the use of suitable calibration curves or the addition of an internal standard (Gros et al. 2006). We used the external calibration curve and internal standards in order to quantify the main hydroxycinnamic acids, flavonols, and anthocyanins in the studied samples. Differences between both methodologies were found, and the results are summarized in Table 3 for external calibration and Table 4 for internal standard.

**Table 3:** Content of main hydroxycinnamic acid (HCA) derivatives, anthocyanins, and flavonol derivatives in *Ribes magellanicum* PEEs (in mg kg^−1^ PEE) quantified by external calibration curve

Compound (peak)	Argentina		Chile	
	Arroyo Casa de Piedra	Laguna Verde, Villa La Angostura	Parque Nacional Conguillio	Reserva Nacional Malalcahuello
HCA derivatives				
**3** a	1826.7 ± 0.9	1602.7 ± 9.5	594.6 ± 0.7	177.8 ± 0.3
**4** a	36.0 ± 0.0	129.4 ± 5.0	ND	ND
**8** a	34.7 ± 0.0	54.0 ± 1.4	33.4 ± 0.9	4.2 ± 0.1
**11** a	99.6 ± 0.2	749.4 ± 17.9	20.3 ± 0.0	86.4 ± 0.3
Anthocyanins				
**17** b	157.5 ± 0.6	ND	275.8 ± 2.3	6.8 ± 0.7
**18** d	241.1 ± 1.0	ND	125.4 ± 0.7	23.1 ± 0.6
**19** d	20.5 ± 0.1	ND	22.0 ± 0.1	ND
**32** d	20.4 ± 0.0	ND	33.0 ± 0.0	ND
**33** b	1.2 ± 0.3	ND	ND	ND
Flavonol derivatives				
**20** c	10.4 ± 0.1	6.3 ± 0.0	5.2 ± 0.0	4.5 ± 0.0
**22** d	105.1 ± 0.0	15.7 ± 0.0	6.2 ± 0.0	6.7 ± 0.0
**37** c	211.9 ± 0.1	76.3 ± 0.1	13.2 ± 0.5	13.6 ± 0.1
**41–43** d	358.4 ± 0.4	97.2 ± 0.0	34.9 ± 0.5	33.3 ± 0.1
**53** d	14.3 ± 0.1	7.0 ± 0.0	5.2 ± 0.1	7.7 ± 0.1
**54** d	44.9 ± 0.2	11.4 ± 0.1	7.1 ± 0.1	7.6 ± 0.1
**56** d	35.7 ± 0.2	18.9 ± 0.1	8.9 ± 0.6	5.7 ± 0.0

PEE, phenolic enriched extract; nd, below quantification limit.

a Expressed as chlorogenic acid equivalents.

b Expressed as cyanidin‐3‐rutinoside equivalent.

Expressed as cyanidin‐3‐glucoside equivalent.

c Expressed as quercetin‐3‐glucoside equivalent.

d Expressed as kaempferol‐3‐glucoside equivalent.

**Table 4:** Content of main hydroxycinnamic acid (HCA) derivatives, anthocyanins, and flavonol derivatives in *Ribes magellanicum* PEEs (in mg kg^−1^ PEE) quantified by internal standard method

Compound (peak)	Argentina		Chile	
	Arroyo Casa de Piedra	Laguna Verde, Villa La Angostura	Parque Nacional Conguillio	Reserva Nacional Malalcahuello
HCA derivatives^a^				
**3**	1864.0 ± 7.8	1592.5 ± 2.0	670.3 ± 28.1	190.2 ± 1.2
**4**	51.6 ± 0.5	183.8 ± 3.5	47.3 ± 2.2	ND
**8**	26.4 ± 1.2	68.3 ± 0.9	53.6 ± 2.4	19.3 ± 0.3
**11**	87.3 ± 0.7	752.7 ± 12.2	44.5 ± 2.4	100.3 ± 0.5
Anthocyanins^b^				
**17**	93.6 ± 1.8	ND	147.7 ± 1.3	9.6 ± 0.3
**18**	199.0 ± 2.3	ND	90.9 ± 0.0	7.9 ± 0.4
**19**	6.6 ± 0.0	ND	8.0 ± 0.1	ND
**32**	6.2 ± 0.0	ND	5.6 ± 0.1	ND
**33**	6.3 ± 0.0	ND	ND	ND
Flavonol derivatives^c^				
**20**	10.2 ± 0.3	5.6 ± 0.1	4.4 ± 0.1	3.5 ± 0.0
**22**	90.4 ± 2.2	13.1 ± 0.2	4.9 ± 0.1	5.3 ± 0.1
**37**	236.8 ± 6.1	82.8 ± 0.3	13.3 ± 0.4	13.8 ± 0.2
**41–43**	309.4 ± 8.2	83.5 ± 0.8	29.7 ± 0.3	28.3 ± 0.8
**53**	11.9 ± 0.2	5.6 ± 0.0	4.1 ± 0.1	6.2 ± 0.0
**54**	38.3 ± 0.7	9.4 ± 0.3	4.8 ± 0.0	6.1 ± 0.0
**56**	30.2 ± 0.6	15.8 ± 0.3	4.6 ± 0.1	4.5 ± 0.0

PEE, phenolic enriched extract; ND, below quantification limit.

a Expressed as *trans*‐caffeic acid equivalents.

b Expressed as delphinidin‐3‐glucoside equivalents.

c Expressed as quercetin‐3‐glucuronide equivalents.

Ripe fruits from Argentina presented a higher content of cyanidin‐3‐glucoside (**18**), whereas in the ripe sample from Chile the rutinoside (**17**) was the main anthocyanin (Table 3 and 4, Fig. 5). Arena & Coronel studied fruit growth and chemical properties of *R. magellanicum* from Ushuaia, Tierra del Fuego, Argentina (Arena and Coronel 2011). Authors described a total anthocyanin content ranging from 0.05 to 2.40 g kg^−1^ fresh fruit weight, increasing during the ripening period. Ruiz et al. (2013) reported a total of 2.44 ± 0.09 *μ*mol of monomeric anthocyanins g^−1^ fresh fruit, whereas by HPLC‐DAD the value was of 5.08 ± 0.01 *μ*mol total anthocyanins g^−1^ fresh fruit. In the red variety of black currants (*Ribes nigrum x pallidum* cv. Red Dutch) only cyanidin derivatives were found in low amounts, whereas in the black variety (*Ribes nigrum* cv. Öjebyn), the main anthocyanins were cyanidin‐3‐rutinoside and delphinidin‐3‐rutinoside (Määttä et al. 2003). The anthocyanin content of the unripe sample from Villa La Angostura, Argentina was below our detection limits. In berries, anthocyanins begin to appear at onset of the ripening stage due to the increase in the expression of genes from the anthocyanin biosynthesis, thus explaining the low amount of anthocyanins in this sample (Ribera et al. 2010; Zhang et al. 2013; Mikulic‐Petkovsek et al. 2015; Veberic et al. 2015). The level of anthocyanins variation has also been related to season, varieties and growing conditions (Bizjak et al. 2013). In *Ribes nigrum* cv. Vertti and *Ribes nigrum x pallidum* cv. White Dutch, the green and white varieties, respectively, anthocyanins were also not detected (Määttä et al. 2003).

Several hydroxycinnamic acids (HCA) have been detected in *Ribes* species (Määttä et al. 2003; Mikulic‐Petkovsek et al. 2015; Ruiz et al. 2015). HCA in *R. magellanicum* fruits from Argentina were about three times higher than in samples from Chile according to both quantification methodologies (Table 3, 4). The main HCA, 3‐caffeoylquinic acid (**3**), was almost 10 times higher in the sample from Arroyo Casa de Piedra compared to the sample collected in the Reserva Nacional Malalcahuello (Table 3 and 4). The different varieties of *R. nigrum* presented as main compounds caffeoyl hexosides derivatives, followed by coumaric acid and ferulic acid derivatives (Määttä et al. 2003). In our analysis the main compounds found were only caffeoylquinic acid derivatives (>90%), followed by lower levels of feruloyl quinic acid and coumaroylquinic acid derivatives. In black currant varieties, 3‐coumaroylquinic acid represented almost 70–80% of total HCA (Mikulic‐Petkovsek et al. 2015). The levels of HCA, as for anthocyanins, vary according to the ripeness stage of the fruit. In apples, Bizjak et al. (2013). demonstrated a decrease by 27% during ripening, whereas MacLean et al. (2006) reported an increase in the level of hydroxycinnamic acids as the fruit maturity progressed. Mikulic‐Petkovsek et al. (2015) proposed that the content levels of individual phenolics groups can be used to predict the optimal ripeness of *Ribes* fruits for harvest. In our samples, the HCA content showed differences between the degree of ripeness and collection place. While in the samples from Argentina the unripe fruit presented higher content than the ripe sample, the ripe fruits from Chile presented higher content than the unripe fruits. However, the results in Argentinean samples are strongly influenced by the particularly high content of feruloylquinic acid (**11**) in the unripe sample.

The main flavonols in *Ribes magellanicum* were quercetin‐3‐glucoside (**37**) and the mixture of quercetin‐hexoside/kaempferol hexoside (**41, 42,** and **43**), in agreement with previous reports (Ruiz 2015). In black currants the major flavonoids are quercetin‐3‐rutinoside, myricetin‐3‐rutinoside, quercetin‐3‐glucoside, and myricetin‐acetylhexoside (Mikulic‐Petkovsek et al. 2015). The samples from Argentina presented higher levels of these compounds (Table 3 and 4). The TF content in *R. magellanicum*, determined by HPLC, was previously reported as 0.68 ± 0.00 *μ*mol g^−1^ fresh fruit (Ruiz et al. 2015).

In food chemistry, the development of chemometrics and multivariate analysis has proven to be a powerful tool in the analysis of a large amount of data (Arvanitoyannis et al. 1999). The multivariate analysis generates mathematical‐statistical models based on quantitative and qualitative information about the natural constituents to obtain characteristic fingerprints of each sample (Geana et al. 2016). The Spearman's rank correlation coefficient was calculated setting the concentration of the identified compounds as the independent variable and the antioxidant activity (DPPH and FRAP) as the dependent variable. Results are presented in Table 5. The most significant correlation was found for compounds **3**,** 20,** and **41–43** and both antioxidant assays. Interestingly, no significant correlation was found among individual anthocyanins and the antioxidant activity. In a study on the correlation of antioxidant capacity (measured as ORAC) and the total phenolic and anthocyanin content in *Vaccinium* species, the authors found that the anthocyanin content of the different blueberry samples was linearly related to the ORAC measurement; however, a better correlation was found among total phenolics and ORAC (Prior et al. 1998). In a study of 26 different berries species, the correlation between the antioxidant activity with flavonols and hydroxycinnamic acids only explained a 31% of total antioxidant activity (Kähkönen et al. 2001). In this study, hydroxycinnamic acid and flavonols seem to be responsible for the antioxidant activity of the *Ribes magellanicum* samples. Nevertheless, according to Liu (2003) this activity is more likely attributed to a synergistic and additive effect of all compounds.

**Table 5:** Correlation coefficients for antioxidant activity (DPPH, FRAP) and the concentration of individual compounds identified in *Ribes magellanicum* samples from Argentina and Chile

Compound	DPPH		FRAP	
	Spearman coefficient	*P* value	Spearman coefficient	*P* value
HCA derivatives				
**3**	−0.958042	0.000001	0.930070	0.000012
**4**	−0.720340	0.008232	0.705411	0.010383
**8**	−0.727273	0.007355	0.769231	0.003446
**11**	−0.580420	0.047856	0.580420	0.047856
Anthocyanins				
**17**	0.070424	0.827831	0.021127	0.948038
**18**	−0.330994	0.293298	0.401418	0.195884
**19**	0.021279	0.947666	−0.014186	0.965099
**32**				
**33**	−0.734316	0.006538	0.752674	0.004728
Flavonol derivatives				
**20**	−0.930070	0.000012	0.930070	0.000012
**22**	−0.797203	0.001900	0.727273	0.007355
**37**	−0.825175	0.000951	0.769231	0.003446
**41–43**	−0.916084	0.000028	0.944056	0.000004
**53**	−0.412587	0.182564	0.363636	0.245265
**54**	−0.762238	0.003950		
**56**	−0.916084	0.000028		

**Figure 6:**
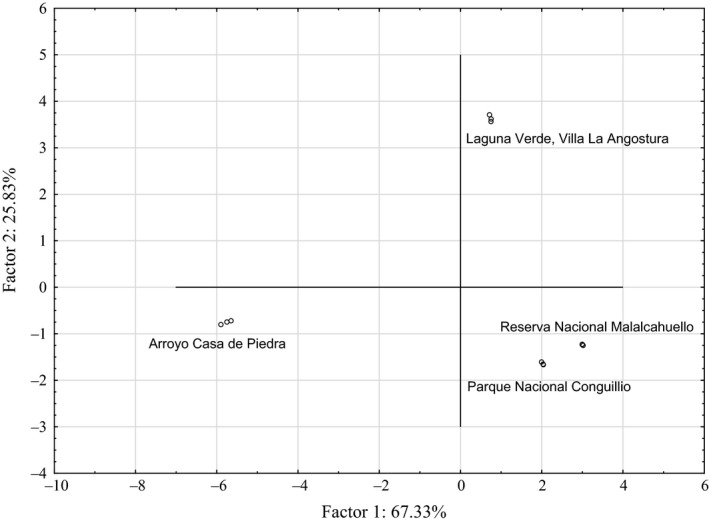
Scatter plot of the first and second discriminating factors showing differences between Argentinean (Laguna Verde, Villa La Angostura, and Arroyo Casa de Piedra) and Chilean (Reserva Nacional Malalcahuello and Parque Nacional Conguillio) *Ribes magellanicum* samples.

Among other chemometric tools, principal component analysis (PCA) is commonly used for discrimination, classification, modeling, and correlation (Di Paola‐Naranjo et al. 2011). As presented in Figure 6, the samples were classified into three clearly separated groups. The first discriminating factor accounts for 67.33% of the variability and the second discriminating factor for 25.83% of the variability. The TF (−0.9966), FRAP (−0.9994), and compounds **33** (−0.9600), **20** (−0.9983), **22** (−0.9868), **37** (−0.9888), **41–43** (−0.9950), **54** (−0.9874), and **56** (−0.9788) showed negative correlation and influenced more significantly the first discriminating factor. On the other hand, compounds **11** (0.9975), **4** (0.9865), and **19** (−0.9899) exerted more effect on the second discriminating factor. This distinction between Chilean and Argentinean *Ribes magellanicum* fruits (Fig. 6) can be considered a first approach; however, more samples are needed in order to obtain a more complete picture.

*Ribes* berries are good sources of anthocyanins, hydroxycinnamic acids, flavonols, and flavan‐3‐ol (Milivojevic et al. 2012), with known biological properties, including in vitro inhibition of enzymes involved in metabolic syndrome, hyperglycemia, and hypertension. Several biological activities have been reported for polyphenols occurring in berries and can explain, at least in part, their positive effect on diet. Inhibition of starch digestion and glucose absorption are some of the possible action mechanisms of polyphenols occurring in foods. Polyphenols inhibit digestive enzymes including *α*‐amylase, maltase, and sucrase. The inhibition of both enzymes in vitro is a method used in studies on the potential beneficial effect of dietary polyphenols. Boath et al. (2012) reported the effect of PEE from black currants (*Ribes nigrum* L. var. 8982‐6) and rowanberry (*Sorbus aucuparia* var. Sahharnaja) on *α*‐glucosidase activity. Both PEEs potentiated the inhibitory effect of acarbose on *α*‐glucosidase and suggest the potential of PEEs from native berries as a complementary dietary mean to control glycemia. The reduction in carbonyl stress and advanced glycation end products has been suggested as a strategy to alleviate diabetic‐related complications. In a recent article by Chen et al. (2014) a *R. nigrum* extract with high anthocyanin content was assessed for its ability to scavenge methylglyoxal (MGO). The blackcurrant had a high content of anthocyanins; both the extract as well as the individual main anthocyanins were able to form MGO adducts. The results suggest that anthocyanin‐rich food sources might prevent diabetic‐related complications through decreasing reactive precursors of *α*‐dicarbonyl/advanced glycation products (Chen et al. 2014). Obesity is a major worldwide problem and changes in diet can contribute to reduction in the intake of fats. The effect of extracts from the berries *Ribes nigrum*,* Vaccinium corymbosum*,* Rubus chamaemorus*,* Rubus stellatus x R. arcticus*,* Vaccinium vitis‐idaea*,* Sorbus aucuparia*,* Fragaria x ananassa* var. Elsanta and *Rubus idaeus* var. Glen Ample on pancreatic lipase was investigated (McDougall et al. 2009). The inhibitory effect of these berries on pancreatic lipase may reduce the rate in which triglycerides are split into absorbable glycerol and fatty acids (de la Garza et al. 2011; Sosnowska et al. 2015). The most active samples as pancreatic lipase inhibitors were *R. stellatus x R. arcticus*,* R. chamaemorus*,* F. x ananassa,* and *R. idaeus*, reducing up to >85% the activity of the enzyme at 50 mg GAE L^−1^. The most active was the raspberry extract with inhibitions up to >95% at the concentration mentioned. Recently, it was reported that anthocyanins from *R. nigrum* were able to attenuate weight gain and improve glucose metabolism in diet‐induced obese mice (Esposito et al. 2015). The results of our study can be useful for further works on *R. magellanicum* fruits from Argentina and Chile, including studies on the inhibition of certain metabolic enzymes that may have potential beneficial health effects.

## Conclusion

In summary, in vitro antioxidant assays showed a higher capacity to scavenge radicals of ripe Argentinean fruits PEE. The most significant cytoprotective effect against oxidative stress‐induced by H_2_O_2_ was found in the sample from Arroyo Casa de Piedra. The Spearman's correlation coefficient pointed out HCA and flavonols as the responsible compounds for the antioxidant activity of the samples. The PCA allowed a preliminary insight about the differences between the Chilean and Argentinean samples. The phenolic compounds identified in the Patagonian currant *Ribes magellanicum* can explain the antioxidant effect found in the samples and suggest further nutraceutical potential for this South American berry.

## Conflict of Interest

None declared.
